# Observations upon the Restoration of Hearing, by Perforating the Tympanum

**Published:** 1809-09

**Authors:** 

**Affiliations:** Garrison Physician at Capel


For some time past, Mr. Astley Cooper's Operation of perforating the Tympanum, in Cases of Deafness, has been either laid aside, or the Operators have ceased to inform the Public of their Success. The following is selected from the Journal Gén. de Medicin, No. 133.

---

*Observations upon the Restoration of Hearing, by perforating the Tympanum.* By Dr. HUNOLD, Garrison Physician at Capel.

M. MICHAELIS having informed Dr. Hunold, by letter, that he had lately succeeded in restoring the faculty of hearing to a lady, by perforating the tympanum, in the manner manner prescribed by Cooper, Dr. Hunold determined to attempt the operation.

Case 1. Ann Catherine Mehlberg, aged 45, deaf for six years past, from a violent inflammation of the ears, occasioned by a severe cold, could not hear at all with her right ear, and could only hear with the left by using an ear trumpet. The right ear was first operated upon, and it was previously cleared of all the wax, &c. until the tympanum, which was white and shining, was plainly discernible. Dr. Hunold pierced it at the lowermost and interior part. The patient instantly heard every thing that was said to her, and informed the operator, that she experienced no pain whatever, but merely a crack, or sharp noise, at the moment of the perforation.

Case 2. M. Wienbrecht, a master locksmith, 50 years of age, was completely deaf in the right ear, and heard very indistinctly with the left. He had lost his hearing at 20 years of age, by falling, when much heated by over exertion, from an eminence into a piece of water, with his head foremost. When taken up, no signs of life remained, and although animation was restored, he was never free from the deafness occasioned by the violent cold he had thus caught, and all the remedies he had subsequently taken proved of no avail. By his own desire, the tympanum of the right ear was perforated, and he instantly heard every thing that was said, even in a whisper.

Case 3. —— Gressin, a widow, 63 years of age, affected from the age of 30, in consequence of blows from her husband, according to her own account, was completely deaf in both ears, which determined Dr. Hunold to operate upon both tympana at once. This patient instantly recovered her hearing perfectly in the left ear, and imperfectly in the right, and was able to repeat, word by word, all that was said to her.

Dr. Hunold has performed about one hundred similar operations, and two-thirds of them have been successful.

*Some*

